# The Cold Case Files of rAAV Capsid Influence on Transduction: New Leads

**DOI:** 10.3390/v17111476

**Published:** 2025-11-05

**Authors:** Sara K. Powell

**Affiliations:** Center for Molecular Medicine, Department of Pediatrics-Metabolism and Genetics, University of North Carolina at Chapel Hill, Chapel Hill, NC 27599, USA; sara_powell@med.unc.edu

**Keywords:** adeno-associated virus (AAV), capsid, transduction, epigenetics, gene therapy

## Abstract

Adeno-associated virus (AAV) is a prevalent vector in viral gene therapy. Given its importance, significant efforts focus on engineering the capsid residues on the exterior surface to increase cell/tissue-specific binding to cellular receptors or to decrease immunogenicity. But there is also a need for stable transgene expression to achieve the desired therapeutic effect. For rAAV transgene regulation, all approaches to date utilize sequence elements within the transgene cassette to restrict the localization and level of transgene expression. However, there is accumulating evidence that rAAV transgene expression can be mediated by rAAV capsid residues distinct from cell receptor binding residues, but these reports have largely gone ‘cold’ due to a lack of a clear mechanism. Thus, these novel rAAV capsid elements may revise interpretation of both past and future rAAV capsid engineering. This review will coalesce the data supporting this novel rAAV capsid role and describe how the capsid influences transgene expression with a focus on interactions with the transgene, binding cellular proteins, and epigenetic modulation.

## 1. Introduction

Adeno-associated virus (AAV) is a single-stranded DNA non-enveloped capsid enclosed *dependoparvovirus* in that it cannot replicate without a helper virus [1]. The AAV genome consists of two genes (*rep* and *cap*) flanked by T-shaped hairpin inverted terminal repeats (ITRs) that serve as origins of replication and are required for genome packaging into assembled capsids. The *rep* gene encodes four proteins (Rep78, Rep68, Rep52, and Rep40) that are expressed during replication, and the *cap* gene encodes three capsid structural proteins, VP1, VP2, and VP3. VP proteins share a common C-terminal sequence while VP1 N-terminal sequence is unique (VP1u) or shared with VP2 (VP1/2). The *cap* gene also encodes two AAV accessory proteins, AAP and MAAP, from alternative reading frames. AAP is the assembly-activating protein that helps assemble capsids [2] and MAAP is the membrane-associated accessory protein [3] that is involved in AAV replication [4,5].

AAV capsids form an icosahedral structure consisting of VP1, VP2, and VP3 protein subunits in an approximate ratio of 1:1:10, respectively. The capsid crystal structures of several AAV serotypes have been resolved, but they only show VP3 subunits ordered [6,7,8] which is similar to other *parvoviruses* [1,9]. While AAV VP1/2 unique regions are not ordered in crystal structures, they are a low resolution mass within the capsid interior which is consistent with their described transduction/infection functions [10]. The interior VP1/2 region is externalized during endosome processing to reveal the phospholipase A_2_ domain (PLA_2_) [11,12] and nuclear localization signal (NLS) [13], both of which are required for AAV transduction/infection [11,13].

**Figure 1:**
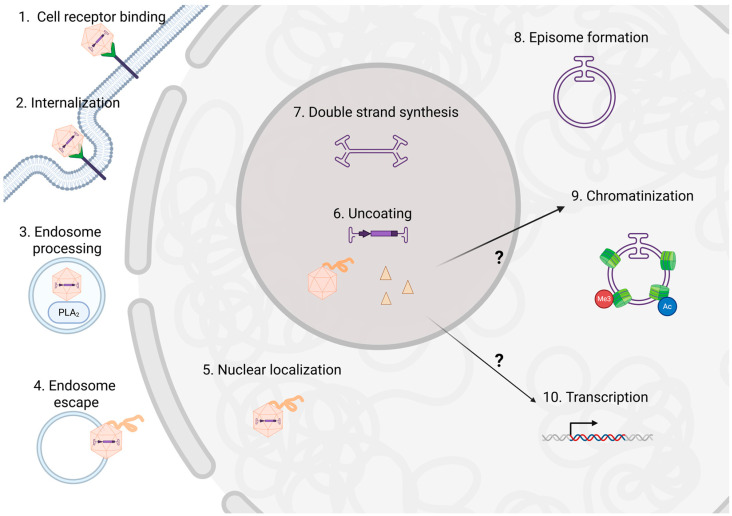
An overview of rAAV transduction. rAAV transduction steps have been thoroughly reviewed elsewhere [18] but is briefly depicted above in numerical order. rAAV binds to cellular attachment factors as well as primary and secondary receptors (step 1) which enable cellular internalization in step 2. rAAV is processed though the endosome where VP1/2 is externalized, exposing the PLA_2_ domain facilitating endosome escape in step 4. The externalization of VP1/2 also exposes nuclear localization sequences (NLSs) to allow for nuclear localization (step 5). The vector localizes the nucleolus to uncoat (step 6) the transgene from the capsid. The fate of the capsid is unknown, but here we review data that suggests it influences transgene expression through transcriptional and/or epigenetic regulation (indicated by ? in the figure). The next step is second strand synthesis (step 7) then the transgenes form episomes (step 8) which become chromatinized (step 9). The final nuclear step is transcription (step 10). Created in BioRender 3.0. Powell, S. (2025) https://BioRender.com (accessed on 23 July 2025).

For recombinant AAV (rAAV) vectors, the wildtype AAV genes (*rep* and *cap*) are required for vector production but are not packaged; instead, a promoter, transgene, and any other regulatory sequence elements within the ITRs are packaged into capsids. The rAAV capsid packages ~4.7 kb of ssDNA and is able to deliver it to a range of cell and tissue types in a capsid serotype-dependent manner [14]. rAAV successfully supports therapeutic transgene expression in humans and several rAAV gene therapy products have gained FDA-approval. Thus, given its importance to the gene therapy field, the rAAV capsid has been widely characterized in terms of cellular receptor binding that are serotype-specific (reviewed in [15]) or more broadly used primary receptors (AAVR [16] and AAVR2 [17]). Further capsid engineering has enhanced desirable features such as increased vector production, cell/tissue specificity, increased stability, decreased immunogenicity, and increased transduction [15]. rAAV transduction consists of several steps, many of which have been assessed to improve overall transduction (Figure 1) through.

rAAV capsid engineering. For example, a rational engineering approach increased transduction and nuclear vector particles by mutation of surface-exposed tyrosine residues to prevent capsid proteasomal degradation [19,20]. However, despite well-reasoned rationales, increased nuclear vector copies were not always met with a similar increase in transduction as measured by transgene mRNA or protein levels. It is also important to note that nuclear entry is not predictive of stable transgene expression because while rAAV2 IV infusion in mice (C57BL/6) results in a substantial amount of hepatocyte nuclei containing rAAV2 transgenes at day 1 post-infusion [21], only 5% of hepatocytes contribute to stable transgene expression [22]. A possible reason for this gap in successful vector nuclear entry and the lack of transgene expression is that the capsid itself plays a role in transgene expression, which is a hypothesis with few solid mechanistic leads through the years, thus remaining a cold case. For example, a mutant rAAV2 trafficked to the nucleus similar to rAAV2 but had reduced transgene expression compared to rAAV2, suggesting a defect in transcription [23]. In another in vitro study, a single amino acid mutation in wtAAV2 had significantly reduced viral mRNA copies despite virus trafficking to the nucleus, uncoating, and exhibiting no defects in second strand synthesis [24]. While rAAV transgene expression is capsid-mediated by several steps during transduction, novel capsid-mediated activities in the nucleus that modulate rAAV transgene expression are becoming more evident. Here, we review how rAAV transgene expression is influenced in a capsid-dependent manner through specific capsid residues, cellular protein binding, and epigenetic regulation with an emphasis on activities within the nuclear compartment. 

## 2. rAAV Capsid in the Nucleus

The rAAV vector enters the nucleus with an intact capsid. rAAV2 capsids are visualized in the nucleus of transduced HeLa cells after 4 h via confocal microscopy using A20 antibody labeling to detect intact capsids [25]. Then, a small fraction of rAAV intact capsids localize to the nucleolus where uncoating can occur. rAAV uncoating rate can influence transgene expression levels which can be serotype-dependent. rAAV2 compared to rAAV6 and rAAV8 demonstrated a slower rate of uncoating, and thus exhibited decreased transduction in the mouse liver [26]. A similar finding was found with rAAV2 and rAAV6 in cardiac cells (PNMCs) in which rAAV2 exhibited slower uncoating and lower transduction, despite similar vector transgene levels within the nucleus. However, rAAV2 exhibited transgene expression earlier than rAAV6, suggesting faster uncoating in HeLa and HMEC cells [27]. Clearly, these findings suggest that uncoating is dependent on both capsid and cellular context but demonstrate that the timing of uncoating of the transgene can influence transgene expression.

The fate of the capsid after uncoating is not entirely known. Studies in vitro suggest that the capsid can disassemble because while wtAAV2 genomes and intact capsids colocalize in nucleoli, so do individual VP subunits and genomes as visualized by immunofluorescent microscopy [28]. However, in vitro studies found two different populations of rAAV8 and rAAV9 capsids as visualized by atomic force microscopy: (1) intact capsids with ejected transgenes, or (2) ruptured capsids due to a temperature-dependent change in capsid structure [29], whether or not these populations are sequential or independent was not pursued. A recent in vitro study suggests they are sequential events because despite differences in capsid stability, wtAAV2 and wtAAV5 capsids disassemble at a higher temperature than seen for genome release, and capsid disassembly occurred after most capsids were empty [30]. Together these studies also illustrate that capsids have different degrees of thermostability, as others have shown as well [31,32], which could be an indication of their rate of uncoating or determine the outcome of the empty capsid. Aside from the data on AAV presented above, data from another *parvovirus* suggests that the genome is ejected from the capsid, but the capsid and genome remain associated with each other [33,34]. Regardless of the AAV capsid’s unknown fate, these results suggest that the capsid or its individual components would still be available to directly influence transgene expression after uncoating in the nucleolus.

## 3. Capsid-Dependent rAAV Transgene Modulation After Nuclear Entry

Given that the capsid is present in the nucleus with an exposed VP1/2 region; the rAAV capsid and particularly the VP1/2 region could possibly influence transgene expression. VP1/2 contains several functional domains identified in silico that are distinct from the PLA_2_ domain and NLS residues [35]. Two such motifs, SH-2 binding (YXXQ) and PDZ-binding, when mutated in wtAAV2 reduced infectivity in HeLa cells. Additionally, the well-conserved S/T motif (_154_DSSS/TG_158_) when mutated reduced rAAV2 transduction and altered in vitro transgene transcription [36]. Further analysis in rAAV2 found that single residue mutations (D154A, S155A, S156A, S157A, and G158A) exhibited the same level of uncoated transgenes in HeLa cells; however, all but S157A exhibited reduced transcript levels. Together this suggests that the S/T motif can alter rAAV transduction at the transcriptional level. Follow-up in vitro studies in rAAV2 and rAAV9 found that mutation of only the core residues (aa155-157) significantly reduced transgene mRNA levels with an overall reduction in transduction. In contrast, mutations in rAAV4 did not elicit the same response. A caveat to these experiments is that rAAV9 already has an alanine at aa157 and transduction studies were performed in different cell types. However, in mice (C57BL/6 female)-only rAAV9 mutations (S155A and S156A) produced discordant differences in rAAV transgenes (total cellular) and transgene mRNA levels in several tissues, brain, heart, lung, liver, spleen, and muscle [37]. Together, these studies suggest that the VP1/2 region of some rAAV capsids can influence transgene expression in some cellular contexts but also highlights a lack of extrapolation of in vitro results to in vivo outcomes.

Prior to studies in rats and NHPs, the observation that the rAAV capsid can impact transgene expression had not been documented in vivo. We found that capsid–promoter interactions influence transgene expression in the central nervous system (CNS) of rats and NHPs [38,39]. rAAV2 and rAAV9 with a constitutive promoter (CBA) driving transgene expression exhibited robust neuronal transduction in the rat CNS. However, an insertion of six glutamates at aa139 (into VP1 and VP2) into both rAAV2 (rAAV2EU) and rAAV9 (rAAV9EU) only altered the cellular tropism of rAAV9 which was skewed to oligodendrocytes, not neurons [38]. In a follow-up study using a different promoter (JeTI) with another mutant consisting of a six alanineinsertion into rAAV9 (rAAV9AU) showed a difference in cellular tropism; rAAV9AU predominantly transduced neurons while the rAAV9 transduced oligodendrocytes [39]. Together this suggested that the rAAV9 VP1/2 capsid region can influence transgene expression in vivo [38,39]. A capsid–promoter interaction was also seen in NHP brain regions with rAAV2-retro with either a CAG or hSyn promoter [39] indicating that rAAV capsids can influence transgene expression with a range of commonly utilized promoters within the CNS.

Further experiments assessed the role of individual rAAV9 VP subunits given that the inserts mutated VP1 (EU), VP2 (EU), and MAAP9 (RRKRRK) in the rat CNS. Both rAAV9 VP1 and VP2 contribute to the capsid–promoter interaction influence on transgene expression while surprisingly, reduced relative transgene mRNA levels were seen with MAAP9 (RRKRRK) [40]. MAAP is an AAV accessory protein expressed during rAAV production [5] and helps vector trafficking to the media [41], without a previously described role in vivo. This novel MAAP9 role was assessed in rAAV9 produced without MAAP9, which resulted in a significant increase in relative transgene mRNA copies without a significant difference in nuclear transgene copies [40]. Taken together, these results indicate that both VP1/2 capsid residues and MAAP can influence rAAV transduction in vivo; however, the mechanism(s) and existence in other tissues are unknown at the time.

Capsid residues outside of VP1/2 can also influence rAAV transgene regulation as shown by several experiments in which nuclear rAAV transgenes levels do not correlate with transgene expression. These observations are particularly striking when the exact same transgene is used with different, but highly similar capsids suggesting a capsid influence.

rAAV8 structural studies identified Y707 within the two-fold interface [42] and the comparable residue mutation Y704A in wtAAV2 results in reduced infection in HeLa cells, but without a significant difference in uncoated nuclear genomes [24]. Further studies with wtAAV2 D529A, K692A, and E562A mutants (within the two-fold interface) found a similar level of uncoated nuclear genomes, but reduced mRNA levels [43]. It should be noted that the previous two studies did not differentiate between VP subunits (all subunits are mutated) and coinfected wtAAV2 and Adenovirus5 [24,43] which has been shown to increase rAAV transduction [44]. Clearly, the rAAV capsid has a role in the transcriptional control of its transgene, but the mechanism(s) is currently unknown. A possible mechanism is that rAAV capsid residues are acted upon by host cellular proteins that result in differential regulation of transgene levels.

## 4. rAAV Capsid-Interacting Proteins That Alter Transduction

As rAAV makes its way through the cell to the nucleus, it can interact with host cellular factors that can influence transgene expression (reviewed in [45]). While the mechanism(s) of how host cellular factors influence the vector is unclear, there is compelling evidence of a capsid-dependent influence. RNF121 (ring finger protein 121; E3 ubiquitin ligase) was found to support rAAV (rAAV1, rAAV2, rAAV6, and rAAV9) in vitro transduction in a capsid-dependent manner [46]. RNF121 overexpression increased transgene expression while knocking RNF121 out decreased rAAV transgene expression. RNF121 suppressed the inhibitory activity of ubiquitin–proteasome pathway factors [46] that ubiquitinate rAAV capsids that resulted in their degradation and reduced transduction [47]. More strikingly, in cells without RNF121, rAAV transgene expression was reduced due to an increase in DNA damage response proteins (VCP/p97; AAA+ ATPase and DNA-PKcs; DNA Protein Kinase) [46], which have been shown to inhibit rAAV transduction (reviewed in [45]), indicating that RNF121 reduced the DNA damage response inhibition of rAAV transduction. While RNF121 and capsid binding was not demonstrated, they do colocalize [46]. Importantly, when RNF121 inhibitory studies were performed using plasmids or unpackaged rAAV transgenes, a reduction in transgene expression was not seen, suggesting that a role for the rAAV capsid and/or transduction process is required for binding or interacting with cellular factors that influence rAAV transgene expression.

Cellular proteins that interact with the rAAV capsid and influence transduction have been identified in high-throughput studies. A protein microarray experiment identified rAAV2 and rAAV8 capsid-interacting proteins as unclassified (30.8%), nucleic acid binding (23.1%), and kinase (17.5%) [48]. In follow-up studies, the rAAV2 and rAAV8 results were validated to show an interaction with CDK2/cyclin A with residues in the N-terminal sequence of the rAAV VP1/2 within basic region 3 (BR3) [48], which serves as an NLS [13]. rAAV2 and rAAV8 both bound to CDK2/cyclin A and inhibited its phosphorylation activity while, conversely, a CDK2/cyclin A inhibitor increased transduction in vitro [48]. It is unclear at what step CDK2/cyclin A inhibits rAAV transduction as only overall transduction was measured.

Another high-throughput study found that rAAV9 capsids interacted with actin/cytoskeletal proteins, RNA-binding proteins, RNA-splicing/processing proteins, chromatin-modifying proteins, intracellular trafficking proteins, and RNA transport proteins [49]. Another group found similar results using a BioID2 proximity labeling approach and found that rAAV8 N-terminal VP1 labeled cellular factors involved in mRNA/RNA metabolic process, nucleic acid metabolic process, DNA transcription, and mRNA processing [50]. Together these studies using a variety of methods and capsids overwhelmingly support the likelihood that proteins involved in DNA/RNA binding and processing interact with rAAV capsids and could possibly act on the rAAV transgene or transcript.

rAAV9 capsid-binding experiments identified two DNA/RNA-binding proteins, namely TDP-43 (transactive response DNA-binding protein 43kDa) and hnRNPA1 (heterogeneous nuclear ribonucleoprotein A1), that were subsequently validated through Western blot [49]. While this study confirmed that these specific DNA/RNA-binding proteins can interact with rAAV capsids, further studies to determine how they and others may influence rAAV transduction were not pursued. Another DNA/RNA-binding protein, PHF5A (PHD finger protein 5A; U2 snRNP complex component) was identified in an siRNA library screen as a restriction factor of rAAV transduction and further experiments suggested an interaction with rAAV2 and rAAV9 [51]. PHF5A, participates in RNA splicing and DNA repair and was found to inhibit in vitro rAAV transduction at a step after second strand synthesis in all serotypes evaluated (rAAV2, rAAV6, rAAV8, and rAAV9). Conversely, when PHF5A expression was inhibited, rAAV transduction and transcripts were increased. Together this suggests that DNA/RNA-binding proteins can bind to rAAV capsids and influence transgene expression. These studies highlight that a variety of cellular proteins can interact with rAAV capsids and influence their transduction, but again the mechanism(s) remains elusive.

## 5. rAAV Capsid Influence on Transgene Chromatinization

Because the wtAAV genome is chromatinized [52,53], it is not surprising that the rAAV transgene can be epigenetically regulated; however, the mechanism(s) is not entirely known. rAAV transgene silencing was first documented in rAAV2 with a CMV promoter driving transgene expression within the CNS. Transgene expression was present seven days post-infusion in the rat olfactory tubercle, caudate, hippocampus, piriform cortex, and inferior colliculus, but there was a marked decrease in transgene expression in the hippocampus and piriform cortex at three months post-infusion, suggesting that the transgene was silenced in certain cellular contexts [54]. This observation was repeated by others with rAAV2 [55] and postulated to be due to DNA methylation as seen in other systems [56]. However, studies have not established the mechanism(s) of rAAV transgene silencing.

rAAV transgene expression has been studied using pharmacological agents to alter histone acetylation which can regulate gene expression; hypoacetylated (histone deacetylases (HDAC) active) genes are associated with lower expression while hyperacetylated (HDAC-inhibited) genes are associated with higher gene expression. Silenced rAAV CMV-driven transgene expression can be reactivated by sodium butyrate (HDAC inhibitor) and Trichostatin A (TSA; HDAC inhibitor) treatment in cultured cells, suggesting that integrated rAAV transgenes were epigenetically regulated in a site-independent manner [57]. Further reactivation of rAAV transgene expression with TSA treatment was only transient and when removed from the media, the transgene was transcriptionally silenced and in a closed chromatin state [58]. Another group found that rAAV2 and rAAV5 in vitro transduction was enhanced in the presence of HDAC inhibitors with changes in epigenetic marks on histone H3 noted [59]. This finding also translated to mice where an increase in rAAV transgene expression in implanted rAAV2 transduced tumors cells occurred in response to HDAC inhibitors. Interestingly and consistent with this study, rAAV9 capsids in mouse brain lysates were found to interact with chromatin modifiers and specifically HDAC4 (histone deacetylase 4) that acts to inhibit rAAV transduction [49]. An increase in rAAV in vitro transgene expression occurs when HDAC4 was reduced. At one assessed time point, HDAC4 reduction also increased rAAV9 capsids detected in the nucleus and colocalization with nucleolin [49], which has been shown to bind wtAAV2 capsids during replication [60]. These studies show that HDAC inhibitors increase rAAV transgene expression. However, it is still unclear how much HDAC inhibition alters the cellular milieu as a contributing factor to enhanced rAAV transduction and/or a potential capsid-binding event to bring the chromatin remodelers to the transgene specifically. These studies will require further studies involving a known binding site and mutation of said residues to determine the mechanism.

Consistent with other results, chromatin remodeling machinery factors inhibit rAAV transgene expression. A series of in vitro studies evaluated rAAV transduction in cells where NP220 (DNA-binding protein), SETDB1 (H3K9 methyltransferase), and HUSH (human-silencing hub) complex members (PPHLN1 (periphilin), TASOR (Transgene activation suppressor), and MPP8 (M-phase phosphoprotein 8)) were knocked out [61]. The HUSH complex has been shown to transcriptionally silence in multiple contexts, but of particular interest is that it has been shown to silence retroviruses [62] with an increase in SETDB1-dependent repressive chromatin marks (H3K9me3) and histone deacetylation [63,64]. In CRISPR/Cas9 depleted NP220, SETDB1, PPHLN1, and MPP8 cells, rAAV2 relative transgene activity was increased to different levels over two time points, suggesting they inhibit rAAV transduction [61]. The absence of NP220 exhibited the most consistent increase in rAAV transduction in which the transgenes were depleted of H3K9me3 repressive marks. At 48 h post-transduction, rAAV2 and rAAV.rh32.33 relative transgene mRNA levels increased, yet there was no significant difference in rAAV5, rAAV6, or rAAV8 despite a significant increase in transgene protein levels. Together these studies show that NP220 acts to inhibit rAAV transduction through transcriptional silencing in a capsid-dependent manner. These studies did not delve into further analysis to find the capsid residues involved in NP220-mediated rAAV transduction inhibition; determining such residues could prove beneficial to improve rAAV transduction in gene therapy products.

A capsid region that exhibits transduction differences through epigenetic modification of the rAAV transgene was identified [50]. An in vitro comparison of two rAAV serotypes (rAAV8 and avian rAAV) packaged with identical transgenes exhibited significant differences in transgene protein levels despite similar nuclear genome copies; avian rAAV was unable to robustly transduce human and mouse cells. The researchers reasoned that the VP1/2 region was responsible given that the early steps of rAAV transduction were not inhibited. To this end, they made a chimeric capsid (avian-8.1) containing the VP1/2 region of rAAV8 (high expression) and the VP3 region of avian rAAV (no expression) then found that avian-8.1 transduced U87 (human glioblastoma) and primary human cardiomyocytes. Subsequent studies found that another chimera, avian-8.3, had an increase in transgene protein levels above avian-8.1, indicating that the N-terminal portion of rAAV8 VP1 was responsible for the rescue in transduction. Interestingly, avian-8.3 transgenes had enrichment of both active (H3K4me3) and inactive (H3K9me3) chromatin marks compared to avian rAAV. These results suggest that the rAAV capsid can mediate chromatinization of its transgene, but the significance of enrichment in both active and inactive chromatin marks on the expressed transgene is unclear.

A similar trend of discordant chromatin marks and transgene expression has also been described in the liver [65]. In vitro studies were undertaken to compare two liver-tropic rAAV capsids (rAAV-LK03 and rAAV-AM) and the epigenetic marks on their respective transgenes. The capsids differ by a single amino acid; rAAV-AM is rAAV-LK03 with a glycine inserted at 265 (G266), but rAAV-AM exhibited a marked increase in transduction in mouse cells. Both capsids were packaged with the same transgene and exhibited similar nuclear transgene copy numbers; however, transgene mRNA and protein levels were significantly higher in rAAV-AM-infected mouse cells in vitro. The rAAV-AM-delivered transgene had an enrichment of active marks over rAAV-LK03, suggesting that the capsid influenced an active chromatin status and thus increased transgene expression when assayed in mouse cells. It should be noted that rAAV-AM, despite robust expression and enrichment of active chromatin marks, also had similar enrichment levels of H3K9me3 (repressive) and H3K27me3 (repressive) as rAAV-LK03 in non-permissive mouse cells. Together these in vitro studies offer insight into the chromatin status of differentially expressed rAAV transgenes, suggesting that transgenes that exhibit robust transgene expression like avian-8.3 [50] and rAAV-AM [65] have an increase in enrichment of active chromatin marks without a significant increase in inactive chromatin marks on their lower-expressing rAAV capsid and transgene counterparts. However, these in vitro studies are limited by the fact that they are unable to convey an in vivo context and time frame applicable to rAAV gene therapy.

The researchers went on to compare rAAV-LK03 and rAAV-AM in mice [65]. A similar difference in transduction as in vitro was seen in mice in vivo at day 3 post-transduction (rAAV-LK03; reduced transduction and rAAV-AM; robust transduction), but there was a significant difference in nuclear transgene copies that could explain the reduction in transgene mRNA and protein levels. Comparing data from day 3 to day 15, the epigenetic status of rAAV-LK03-delivered transgene changed in enrichment of core histones and both active and inactive histone marks, suggesting the epigenetic marks were not stable and therefore not indicative of transgene expression levels. Thus, in contrast to the in vitro data, rAAV-LK03-delivered transgenes in mouse liver were enriched with both active and inactive chromatin marks on day 15 while exhibiting a lower transgene protein level. The role of the capsid in the transgene epigenetic status is striking, but the contradictory chromatin marks at the assayed time points suggest more data is needed to understand the significance (if any) of histone marks and obtaining stable rAAV transgene expression. rAAV-stable transgene chromatin has been observed after intramuscular injections into NHPs such that rAAV1 transgene expression was not altered by a pharmacological HDAC inhibitor administered over 3 months at 5.5 months post-injection in the one animal tested [66]. While these studies were performed in different contexts and the latter did not assess specific chromatin marks; studies assessing the pathway to stable transgene expression and chromatin marks have not been performed to date. Given that the capsid can influence the epigenetic marks of its transgene, it is important in our understanding of rAAV gene therapy products.

## 6. Conclusions

The rAAV capsid can influence the transduction of its packaged transgene in a manner that is distinct from the established role in cellular transduction. The mechanism(s) of capsid-mediated transgene regulation is unclear. This review covers several instances where the capsid exerts an influence on transgene expression, uncoating, transgene mRNA level, promoter interaction, cellular factor interaction, or chromatin marks, which could act mutually exclusively or inclusively (summarized in Table 1 and Table 2).

While specific and consequential capsid residues (Table 1) that have been shown to influence transgene expression are discussed, the mechanism could be related to overall capsid stability. Differences in capsid stability clearly plays a role in uncoating at different rates [26,27] but strikingly, uncoated vectors have been detected in the nucleus [67] even years after administration [68]. Even more striking is that two different transgenes can form concatemers, when administered a week apart [21], suggesting that uncoating can indeed happen at different rates but does not prevent future transgene processing steps.

How the rate of uncoating translates, if at all, to the coveted stable long-term transgene expression is unclear. Stable transgene expression likely involves a stable chromatin state that remains unaltered. For example, when rAAV-administered animals were dosed with sodium phenylbutyrate (HDAC inhibitor), no change in transgene expression was seen [66]. The capsid has been shown to influence transgene epigenetic marks, but further study is required to determine direct capsid interactions on the formation of a specific chromatin state. However, studies in vivo looking at the chromatin state of the same transgene that were differentially expressed in a capsid-dependent manner [65] suggests a more nuanced progression to gain stable transgene expression [66]. These in vivo studies demonstrate a changing state of active and inactive chromatin marks that were not correlated with transgene expression [65]. However, a caveat to these studies is that they were limited to early time points (day 3 and day 15) that were perhaps too early for the establishment of stable chromatin. However, these studies could also indicate that the inability of a capsid to stabilize transgene chromatin marks relates to transgene expression. The mechanism(s) of how a capsid influences the chromatin state of its delivered transgene is unknown but would certainly be a transformative innovation in the rAAV gene therapy field.

**Figure 2:**
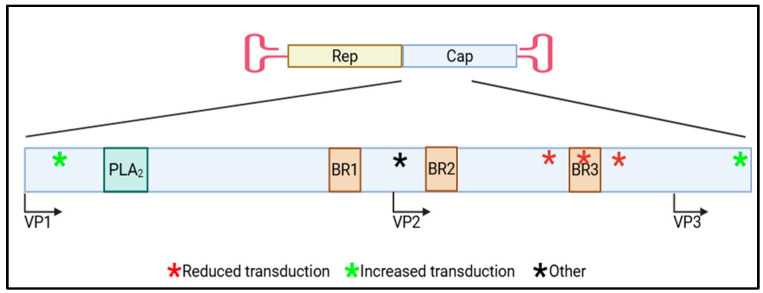
Cartoon diagram of rAAV capsid VP sequence with VP1/2 regions that influence rAAV transduction marked (*). The indicated portion of the *cap* gene sequence is enlarged to show the approximate locations of the VP1, VP2, and VP3 protein start codons, rAAV functional domains (PLA_2_ and BR1-3), and regions that influence transduction (* indicates approximate location with the color corresponding the effect on transduction (red (reduced), green (increased), or black (other)). Created in BioRender 3.0. Powell, S. (2025) https://BioRender.com (accessed on 23 July 2025).

Given that VP1/2 makes up only about a quarter of the VP sequence, it is striking that residues within VP1/2 have been overwhelmingly implicated to influence transgene expression (Figure 2). However, these residues are only within the capsid during early rAAV transduction steps which makes them available for rAAV after cellular entry transduction steps, which is a technique already employed by rAAV to expose its PLA_2_ domain to enable the next step in transduction. The newly exposed VP1/2 would then be able to interact and bind to cellular factors that could be retained until the uncoating step when then they could interact with the transgene and influence expression. Another possible mechanism is that the cellular factors alter the newly exposed VP1/2 in a manner to influence transgene expression (i.e., post-translational modifications). To begin to capitalize on the findings discussed, further investigation is needed to determine commonalities in both capsid, transgene (promoter), and cellular contexts. Undoubtedly further studies are required to define the mechanism(s), but this region of rAAV capsids offers added opportunities to mediate transgene expression beyond VP3 engineering. rAAV gene therapy can become more efficient with these combined rAAV engineering efforts to modulate an appropriate transgene expression level.

## Figures and Tables

**Table 1:** Summary of AAV capsid residues implicated in influencing expression. Each AAV capsid and its associated mutation/residue that led to the documented effect is noted with the respective reference. All amino acid (aa) residues use VP1 numbering.

Capsid	Mutation/Residues(s)	Effect	Ref
rAAV2	A167K	Reduced rAAV transduction	[23]
wtAAV2	Y704A	Reduced wtAAV transduction	[24]
wtAAV2	D529A, K692A, and E562A individually	Reduced wtAAV transduction	[43]
rAAV2	D154A, S155A, S156A, and G158A individually	Reduced rAAV transduction	[36]
rAAV2	S155A, S156A, and S157A together	Reduced rAAV transduction	[37]
rAAV2	PARKRL (BR3)	Reduced rAAV transduction	[48]
rAAV8	PARKRL (BR3)	Reduced rAAV transduction	[48]
rAAV9	S155A and S156A together	Reduced rAAV transduction	[37]
rAAV9	S155A and S156A together	Reduced rAAV transduction	[37]
rAAV9	6 glutamate inserts at aa139	Altered rAAV cellular tropism	[38]
rAAV9	6 alanine inserts at aa139	Altered rAAV cellular tropism	[39]
rAAV2-retro		Altered rAAV cellular tropism	[39]
Avian rAAV	rAAV8 VP1/2 (avian-8.1)	Increased rAAV transduction	[50]
Avian rAAV	N-terminal rAAV8 VP1 (avian-8.3)	Increased rAAV transduction	[50]
rAAV-LK03	G266 = rAAV − AM	Increased rAAV transduction	[65]

**Table 2:** Summary of rAAV capsid-interacting proteins that have been shown to bind or alter rAAV transduction. Each rAAV capsid and documented protein with its inferred function on rAAV transduction is noted with the respective reference.

Capsid(s)	Protein	Function	Ref
rAAV1, rAAV2, rAAV6, and rAAV9	RNF121 (Ring finger protein 121)	Supports rAAV transduction	[46]
rAAV2 and rAAV8	CDK2/cyclinA	Inhibits rAAV transduction	[48]
rAAV9	TDP-43 (transactive response DNA-binding protein 43kDa)	Binding validated by Western blot	[49]
rAAV9	hnRNPA1 (heterogeneous nuclear ribonucleoprotein A1)	Binding validated by Western blot	[49]
rAAV9	HDAC4 (histone deacetylase 4)	Inhibits rAAV transduction	[49]
rAAV2, rAAV6, rAAV8, and rAAV9	PHF5A (PHD finger protein 5A)	Inhibits rAAV transduction	[51]
rAAV2, rAAV5, rAAV6, rAAV8, and rAAV.rh32.33	NP220 (DNA-binding protein)	Inhibits rAAV transduction (discordant transgene mRNA and protein levels)	[61]
rAAV2	SETDB1 (H3K9 methyltransferase)	Inhibits rAAV transduction (under certain conditions)	[61]
rAAV2	PPHLN1 (periphilin)	Inhibits rAAV transduction (under certain conditions)	[61]
rAAV2	MPP8 (M-phase phosphoprotein 8)	Inhibits rAAV transduction (under certain conditions)	[61]

## Data Availability

No new data were created or analyzed in this study. Data sharing is not applicable to this article.

## Abbreviations

The following abbreviations are used in this manuscript:

aa	Amino acid
AAP	Assembly-activating protein
AAV	Adeno-associated virus
Ad5	Adenovirus 5
BR3	Basic region 3
CAG	Chicken β-actin promoter with CMV enhancer and rabbit β-globin slice acceptor site
CBA	Chicken β-actin promoter with hybrid CMV enhancer
CBh	Chicken β-actin hybrid promoter with MVM intron
CDK2	Cyclin-dependent kinase 2
CMV	Cytomegalovirus
CNS	Central nervous system
DNA-PKcs	DNA protein kinases
HDAC	Histone deacetylase
hnRNPA1	Heterogeneous nuclear ribonucleoprotein A1
hSyn	Human synapsin 1 promoter
HUSH	Human silencing hub
JeTI	Synthetic promoter with intron
MAAP	Membrane-associated accessory protein
MPP8	M-phase phosphoprotein 8
NHP	Non-human primate
NLS	Nuclear localization signal
NP220	Nuclear protein 220
PHF5A	PHD finger protein 5A
PHD	Plant homeodomain
PLA2	Phospholipase A2
PPHLN1	Periphilin 1
RNF121	Ring finger protein 121
SETDB1	SET domain bifurcated 1
SH2	Src homology 2
TASOR	Transgene activation suppressor protein
TDP-43	Transactive response DNA-binding protein 43kDa
TSA	Trichostatin A
VCP/p97	Valosin-containing protein
VP	Viral protein
